# 

**Published:** 1857-08

**Authors:** 


### SUBSCRIPTIONS RECEIVED.

Drs. W. E. Wilson, Ill., 1st and 2d vols.; C. R Moore, Ga., 2d vol.; John Rambo, Ga., 1st and 2d vols.; S. C. Hitchcock, Ga., 2d vol.; H. H. Johnson, Ga., 1st vol.; Wm. Booth, Fla., 2d and 3d vols.; J. J. Scott, Miss., 2d vol.; T. T. Mounger, Fla., 2d vol.; Dean & Dupree, Ga., 1st and 2d vols.; N. B. Drewry, Ga., 2d vol.; J. C. Story, Ala., 2d vol.; J. W. Kinnebrew, Ga., 1st vol.; John Rhea, Ga., 2d vol.; Wm. McLean, Ga., 2d vol.; S. H. Freeman, Ga., 2d vol.; Wm. Booth, Fla., 1st and 2d vols.; J. B. Forbes, Ga., 1st and 2d vols.

### TO CORRESPONDENTS.

☞ All Communications pertaining to the Editorial Department of the Journal, should be addressed to the Editors.

☞ All Communications, having reference to the Subscription, the Advertising or the Financial Department, should be addressed to the Treasurer, J. G. Westmoreland, M. D., Dean of the Faculty of the Atlanta Medical College.

